# Oxidative Stress, Persistent Inflammation and Blood Coagulation Alterations in Serum Proteome of Patients with Neovascular Age-Related Macular Degeneration

**DOI:** 10.3390/life14050624

**Published:** 2024-05-12

**Authors:** Mateusz Winiarczyk, Bernd Thiede, Tor Paaske Utheim, Kai Kaarniranta, Dagmara Winiarczyk, Katarzyna Michalak, Jerzy Mackiewicz

**Affiliations:** 1 Department of Vitreoretinal Surgery, Medical University of Lublin, 20-079 Lublin, Poland; jerzymackiewicz@umlub.pl; 2 Department of Biosciences, University of Oslo, 0371 Oslo, Norway; bernd.thiede@ibv.uio.no; 3 Department of Medical Biochemistry, Oslo University Hospital, 0372 Oslo, Norway; uxutto@ous-hf.no; 4 Department of Ophthalmology, Oslo University Hospital, 0450 Oslo, Norway; 5 Department of Ophthalmology, University of Eastern Finland, 70211 Kuopio, Finland; kai.kaarniranta@uef.fi; 6 Department of Ophthalmology, Kuopio University Hospital, 70200 Kuopio, Finland; 7 Department of Molecular Genetics, University of Lodz, 90-136 Lodz, Poland; 8 Department and Clinic of Animal Internal Diseases, Faculty of Veterinary Medicine, University of Life Sciences, 20-612 Lublin, Poland; dagmara.winiarczyk@up.lublin.pl; 9 Department of Epizootiology and Clinic of Infectious Diseases, University of Life Sciences, 20-612 Lublin, Poland; katarzyna.michalak@up.lublin.pl

**Keywords:** AMD proteome, proteomics, serum biomarkers, age-related macular degeneration

## Abstract

Neovascular age-related macular degeneration (AMD) is a major cause of irreversible blindness in elderly populations in developed countries. AMD’s etiopathology is multifactorial, with strong environmental and genetic components, but the exact molecular pathomechanisms underlying the disease are still unknown. In this study, we analyzed blood serum collected from 74 neovascular AMD patients and 58 healthy controls to identify proteins that may serve as potential biomarkers and expand our knowledge about the etiopathogenesis of the disease. The study revealed 17 differentially expressed proteins—11 up-regulated and 6 down-regulated—in neovascular AMD, which are involved in the biological processes previously linked with the disease—oxidative stress and persistent inflammation, impaired cellular transport, lipid metabolism and blood coagulation. In conclusion, the differences in the expressions of the proteins identified in this study may contribute to our understanding of the mechanisms underlying AMD and possibly serve in future as promising biomarkers.

## 1. Introduction

Age-related macular degeneration (AMD) is a progressive, usually bilateral disease affecting the macula—the central part of the retina that is responsible for central, high-quality vision in good-lighting conditions. AMD is usually divided into its atrophic (dry) form, where retinal pigment epithelium (RPE) cells suffer progressive loss, and neovascular (wet, nAMD), with the creation of the pathological choroidal neovascularization (CNV) sprouting from the choroid into the subretinal or retinal tissue, with a tendency to leakage and evoking detrimental oedema. Both the atrophic and neovascular forms share some hallmarks, like the presence of the drusen—subretinal debris deposits. While numerous clinical trials are ongoing, there is currently no effective treatment for dry AMD. In nAMD, anti-vascular endothelial growth factor (anti-VEGF) intravitreal injections are the main line of treatment, yet their effect is usually limited to stopping or slowing down the disease, less often to significant visual improvement, especially if the treatment is introduced in the advanced form of the disease.

According to the WHO, the global population is rapidly aging, and the prevalence of AMD is therefore expected to rise. The estimated global prevalence of AMD in a population aged between 45 and 85 is 8.69%, and an increase from 196 to 288 million cases is expected from 2020 to 2040 [1]. Early diagnosis enables adequate treatment and a better prognosis. As such, there is a strong need for early screening strategies and better understanding of the disease on the molecular level.

The etiology of AMD is not fully understood, yet it is known to be a multifactorial disease, with strong genetic and environmental risk factors, where chronic oxidative stress, impaired clearance and prolonged inflammation result in pathological choroidal neovascularization [2,3]. A number of proteomic studies on AMD have already been conducted, with sometimes conflicting results, yet there are some patterns in the identified proteins and the metabolic pathways that they are involved in that support our current knowledge of the disease’s etiology [4,5,6,7]. In our previous studies, we focused on the tear film proteome, providing interesting findings [8,9,10]. In this study, we aim to characterize the serum proteome of nAMD patients and discuss the potential clinical value of the obtained results.

## 2. Materials and Methods

After fulfilling the inclusion criteria, 132 age- and gender-matched patients were included in the study. The control group consisted of 58 people, and the AMD group consisted of 74 patients. The control group consisted of patients scheduled for cataract surgery who showed no other ocular pathology. The AMD group consisted of advanced neovascular AMD patients receiving ant-VEGF treatment, all in the active phase of the disease. The control group consisted of 28 men and 30 women, with a mean age of 73.51 (SD = 7.99) years, while the study group consisted of 34 men and 40 women, with a mean age of 73.91 (SD = 9.36). The inclusion criteria for the AMD group were as follows: an active form of disease featuring choroidal neovascularization (CNV) on fluorescein angiography or angio-OCT in at least one eye and the presence of subretinal or intraretinal fluid.

The exclusion criteria were any ocular diseases that would disturb the results, e.g., diabetic retinopathy, glaucoma and previous ocular surgery except for cataract extraction. Additionally, any moderately advanced or advanced stage of any systemic disease, such as diabetes, hyperlipidemia, uncontrolled hypertension, cardiovascular disease or autoimmune disorder, was an exclusion criterion.

### 2.1. In-Solution Digestion

To start, 50 µL 25 mM ammonium bicarbonate, pH 7.8, was added to a 3 µL serum sample. For the reduction and alkylation of cysteines, 2.5 µL of 200 mM DTT in 100 mM Tris-HCl, pH 8, was added, and the samples were incubated at 37 °C for 1 h, followed by the addition of 7.5 µL 200 mM iodoacetamide for 1 h at room temperature in the dark. The alkylation reaction was quenched by adding 10 µL 200 mM DTT at 37 °C for 1 h. Subsequently, the proteins were digested with 5 µg trypsin GOLD (Promega, Madison, WI, USA) for 16 h at 37 °C. The digestion was stopped by adding 5 µL 50% formic acid, and the generated peptides were purified using a 10 µL OMIX C18 micro-SPE pipette tip (Agilent, Santa Clara, CA, USA) and dried using a Speed Vac concentrator (Concentrator Plus, Eppendorf, Hamburg, Germany).

### 2.2. LC-MS Analysis

The samples were analyzed by LC-MS using a timsTOF Pro (Bruker Daltonik, Bremen, Germany) which was coupled online to a nanoElute nanoflow liquid chromatography system (Bruker Daltonik, Bremen, Germany) via a CaptiveSpray nanoelectrospray ion source. The dried peptides were dissolved in 4 µL 0.1% formic acid, and 2 µL of sample was injected. The peptides were separated on a reversed-phase C18 column (25 cm × 75 µm, 1.5 µm, PepSep (Bruker Daltonics, Bremen, Germany)). Mobile phase A contained water with 0.1% formic acid, and acetonitrile with 0.1% formic acid was used as mobile phase B. The peptides were separated by a gradient from 0 to 35% mobile phase B over 30 min at a flow rate of 300 nl/min at a column temperature of 50 °C. MS acquisition was performed in DDA-PASEF mode. The capillary voltage was set to 1.5 kV, with a mass range of 100 to 1700 m/z. The number of PASEF ranges was set to 20, with a total cycle time of 1.16 s, charge up to 5, target intensity of 20,000, intensity threshold of 1750, and active exclusion with release after 0.4 min. An inversed reduced TIMS mobility (1/*k*_0_) of 0.85–1.40 Vs/cm^2^ was used with a time range of 100 ms, an accumulation time of 100 ms, a duty cycle of 100%, and a ramp rate of 9.51 Hz. Precursors for data-dependent acquisition were fragmented with an ion-mobility-dependent collision energy, which was linearly increased from 20 to 59 eV.

### 2.3. Database Search

The LC/MS data were searched against the human Uniprot database (20,384 entries) using PEAKS X+ software version 10.5 (Bioinformatics Solutions, Waterloo, ON, Canada). The following parameters were used: digestion enzyme, trypsin; maximum missed cleavage, 2; fragment ion mass error tolerance, 0.03 Da; and parent ion error tolerance, 15 ppm. The oxidation of methionine and acetylation of the N-terminus were specified as variable modifications and carbamidomethylation of cysteines as a fixed modification. The maximum number of PTMs per peptide was set to 2. A false discovery rate of 1% was applied to the datasets.

### 2.4. Label-Free Quantification

For label-free quantification (LFQ) using PEAKS, ID-directed LFQ with outlier removal was applied. The following parameters were used on the peptide features: quality ≥ 3, peptide ID count per group ≥ 1, detected in at least ten samples per group. The following parameters were applied for the protein: FDR ≤ 1%, fold change ≥ 2, significance method ANOVA with at least 2 peptides.

## 3. Results

**Figure 1:**
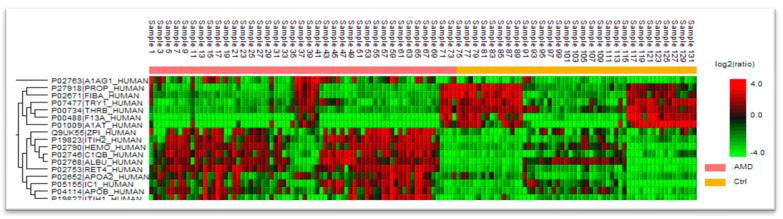
Distribution of proteins in groups with given ratio.

**Figure 2:**
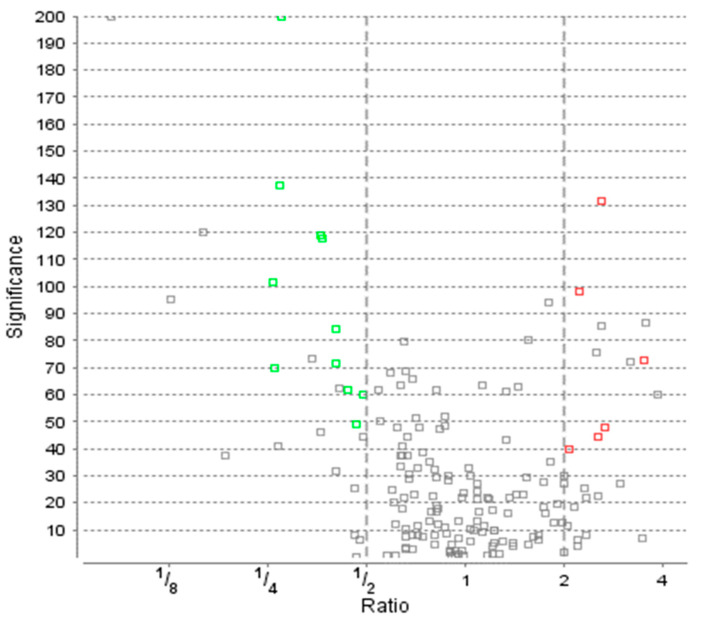
Protein distribution with ratio and significance according to ANOVA analysis of Variance. AMD—age-related macular degeneration study group; Ctrl—control group. Green squares represent the significantly upregulated proteins in AMD group while red the downregulated ones. Grey color means the proteins did not satisfy other conditions.

**Figure 3:**
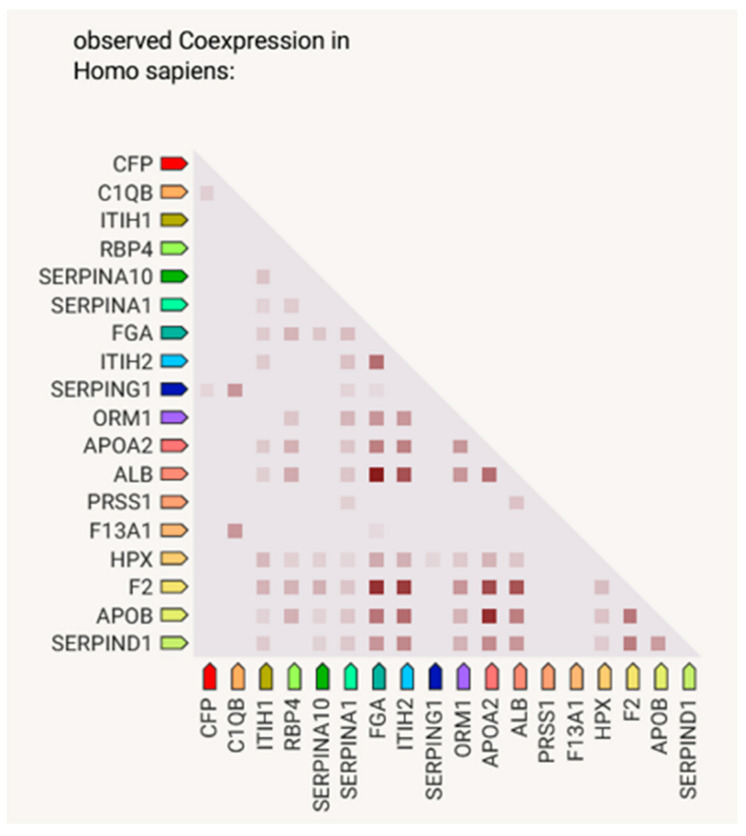
Gene-of-interest co-expression, created using STRING. Lighter and darker marks represent weaker and stronger co-expression scores, based on RNA expression patterns and protein coregulation given by ProteomeHD.

**Figure 4:**
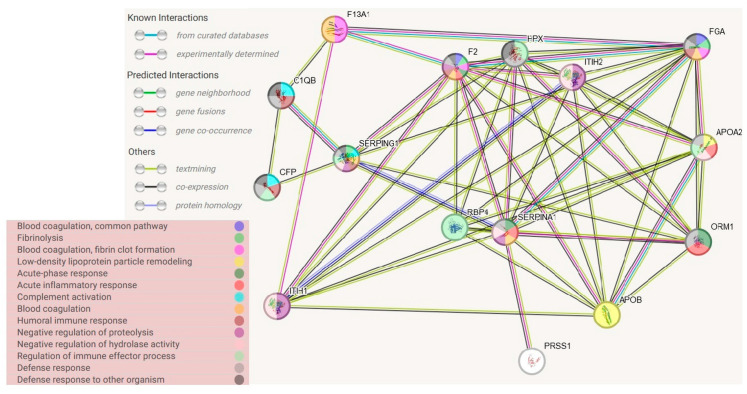
Protein–protein association networks, determined using STRING database. Abbreviations refer to protein names in Uniprot database.

Blood serum collected from 74 late-stage, neovascular AMD patients and 58 healthy controls were analyzed by liquid chromatography coupled with mass spectrometry (LC-MS)-based proteomics. In total, 748 protein groups were identified (Figure 1). The label-free quantification revealed 17 differentially expressed proteins comparing the blood serum of both groups. There were 11 up-regulated proteins in the AMD group (Table 1 and Figure 2), whereas 6 were down-regulated (Table 2). The main functions and common pathways of different proteins were determined using the STRING database, powered by the Swiss Institute of Bioinformatics, Novo Nordisk Foundation Center for Protein Research and European Molecular Biology Laboratory [11]. This application allows us to predict and check direct (physical) and indirect (functional) associations between proteins and to predict protein–protein interactions. The gene-of-interest co-expression is presented in Figure 3, while the protein–protein association network is visualized in Figure 4. The main functions of the identified proteins according to the STRING database are summarized in Table 3.

## 4. Discussion

In this study, we identified 17 different proteins, 11 up-regulated and 6 down-regulated, in neovascular AMD. They were categorized into groups depending on their involvement in metabolic pathways and biological processes, as further described below.

In the course of the STRING connection analysis between the identified proteins, 72 edges were observed, representing a variety of protein–protein interactions, including gene fusion or structural homology (Figure 3). Additionally, the functionalities of proteins were also determined and presented by color (Figure 4, Table 3). The main biological process that was represented by the majority of proteins relates to the defense response, correlated with the immune status, or binding and transporting particles such as ligands, small proteins or ions. To observe more correlations between proteins, a diagram representing co-expression was created to capture the relationships between proteins that do not physically interact or colocalize. Their genes correlated in expression across a large number of experiments [12]. The various types of interrelationships between the proteins may be a valuable starting point for to further research on the proteins identified in this publication.

### 4.1. Impaired Cellular Transportation

Maintaining homeostasis in retinal tissue is extremely demanding for the RPE cells, as they are some of the most metabolically active cells in the human organism [13]. A constant need to excrete the waste material is supported by a highly complex system of influx–outflux transportation and energy delivery [14]. With aging, this system starts to collapse, resulting in the accumulation of waste material in the form of drusen on the border of Bruch’s Membrane (BM) and RPE [15,16,17]. Disruptions in the communication between cells and the breakdown of cellular waste removal systems, particularly autophagy, have been observed in AMD. RPE cells deteriorate with age, reducing their capability to clear metabolic by-products, resulting in the buildup of clumped, misfolded proteins outside cells due to the failure of autophagy—a recurring theme across various neurodegenerative disorders like Parkinson’s disease or Alzheimer’s disease [2,18,19].

In this study, we found the altered levels of proteins involved in the transportation process, which may reflect its breakdown in AMD.

Retinol-binding protein 4 (RBP4) was the most profoundly up-regulated protein in our study, with a 4:1 ratio in AMD patients. This low molecular protein creates a complex with transthyretin (TTR), maintaining the serum retinol (vitamin A form) in the bloodstream, and is solely responsible for delivering it into the RPE cells. Meanwhile, in RPE cells, all-trans retinol accelerates the formation of drusen with lipofuscin formed by A2E accretion. There are reports of up-regulation of RBP4 in serum in the course of AMD, and drugs being developed to limit its expression, leading to a lower RBP4-mediated lipofuscin bisretinoid distribution into the retina, where they are involved in lipofuscin aggregation in RPE cells [20].

RBP4 has mostly been linked in ophthalmology with the atrophic form of AMD and Stargardt disease [20,21,22]. There are preliminary data on a phase 1b/2 clinical trial of Tinlarebant—an oral RBP4 antagonist, showing its potential in slowing or preventing new lesions in Stargardt disease [23]. Given the natural course of the disease, with a formation of the atrophic scar also occurring in the neovascular form, it can be hypothesized that RBP4 is also involved in late-stage nAMD.

Other transportation proteins, described in more detail below, were albumin, alpha-1- acid glycoprotein 1, hemopexin, apolipoprotein A-II and B and Inter-alpha-trypsin inhibitors 1 and 2.

### 4.2. Trypsin Metabolism

Trypsin is a serine protease secreted by the pancreas, with the main function of proteolysis in the small intestine. It has three major isoenzymes (Trypsin-1, Trypsin-2, Trypsin-3), each with a different preference for cleavage of lysine and arginine [24]. Its alterations have not been previously linked to the development of AMD.

Alpha-1-antitrypsin, a serpin and one of the major trypsin inhibitors, was previously found to be up-regulated in the vitreous body of nAMD patients [25], as well as in the aqueous and blood of patients with glaucoma [26]. It is an acute phase protein inhibiting numerous proteases and therefore playing a protective role in the inflammatory process.

Inter-alpha-trypsin inhibitors (ITIs) consist of proteins with high inhibitory action against trypsin. ITI heavy chain 2 was previously found to be up-regulated in dry AMD, where its role as a hyaluronan carrier in serum was underlined. As hyaluronan is involved in extracellular matrix (ECM) remodeling and inflammation, the up-regulation of ITIH2 was seen as a risk factor for AMD progression [27,28]. ITIH2 was also proposed as a potential biomarker of tuberculosis and was considered as a tumor growth inhibitor when overexpressed [29,30].

We noticed ITIH1 and ITIH2 to be almost three- and four-fold up-regulated in the nAMD patients, with simultaneous down-regulation of Alpha-1-antitrypsin and Trypsin-1. These findings may suggest suppression in the trypsin metabolism in the course of AMD.

### 4.3. Coagulation Balance

Although previous studies have shown that coagulation factors probably do not play a significant role in AMD [31,32,33], there are conflicting data on anti-VEGF injections’ effect on the coagulation parameters [34].

Prothrombin is a serum glycoprotein, an inactive thrombin form. Prothrombin time (PT) is a parameter that is used clinically for the evaluation of the clotting time. Coagulation factor XIII A (F13A1) is converted into an active form by thrombin, being involved in the coagulation balance by stabilizing the thrombus [35]. Fibrinogen in the final stage of coagulation is converted into fibrin via thrombin-mediated proteolytic cleavage. It is also an acute phase protein (APP), elevated in previous studies concerning AMD [3,36,37,38,39].

Both PT and F13A1 gene polymorphisms were previously associated with the response to photodynamic therapy (PDT) in nAMD [40]. As PDT is currently almost completely displaced by anti-VEGF therapy, no follow-up study has been performed.

Protein Z-dependent protease inhibitor (ZPI) is a protein from the serpin family, with a major anti-coagulation effect by the inhibition of factor Xa in the presence of protein Z (PZ). In healthy subjects, ZPI and ZP usually circulate as a complex [41,42]. Elevated levels of protein Z were found in central retinal and vein occlusion [43], and ZPI was expressed with a significant difference in nAMD in another plasma proteomic study [44].

All the coagulation balance proteins were significantly down-regulated in our study, except for protein Z-dependent protease inhibitor, which is in line with the findings of the study by Altinkaynak et al. [34], probably being the effect of numerous intravitreal aflibercept injections. Blood coagulation is still being investigated in nAMD, as hemorrhagic events usually dramatically worsen the course of the disease, leaving large retinal scars, with no prognosis for functional improvement. Whether the disturbance of the coagulation protein levels in our study is a part of the etiopathology of nAMD, or the effect of anti-VEGF injections, is unclear. Either way, it seems that proper regulation of coagulation disorders would be beneficial in AMD patients, especially in the group at high risk of developing hemorrhagic events.

### 4.4. Lipid Metabolism

Lipid metabolism is crucial for the pathogenesis of AMD. It is hypothesized that the activity and composition of high-density lipoproteins (HDLs), together with other lipid disorders, are factors in AMD’s pathogenesis, as lipids are a major component in drusen. Similarities were suggested in the pathophysiology of AMD and cardiovascular disease [45]. Also, as was established by the AREDS reports, a number of available dietary supplements are rich in omega 3, 6 and polyunsaturated fatty acids (PUFAs), and it seems as though it is not the high total lipids that are responsible for AMD, but more so the prevalence of the “bad” lipids (TG, LDLs) and an impaired metabolism on the cellular level of RPE, especially given that RPE cells are the most metabolically active body cells—thus, even minor alterations on a cellular level can cause serious detrimental effects [46]. Also, as elevated total lipids are generally known to increase the risk of cardiovascular diseases, patients are usually aware of the need for lipid control. There is also a new concept of ferroptosis being one of the crucial mechanisms of cell death in AMD [47]. It is initiated by lipid peroxidation and iron-dependent accumulation in RPE cells. This can be linked with higher hemopexin levels in our AMD group, as discussed further.

APOA2 mediates the cholesterol transferring into high-density lipid C (HDL-C), together with apolipoprotein A-I (APOA1), helping the RPE cells eliminate the lipidic debris. Both APOA1 and APOA2 are discussed as biomarkers and treatment targets in diabetic retinopathy (DR), polypoidal choroidal vasculopathy (PCV), and AMD, although there is no consistency in studies regarding the mechanism of action [48,49,50,51].

ApoB-100 is a protein that is secreted by the RPE cells, and it was found to be a major early component in drusen formation [52,53]. It was recently suggested that apoB100 plays a protective role in the development of CNV [54]. In the study by Cao et al., ApoB-100 was more up-regulated in the aqueous humor of patients with early and intermediate non-neovascular AMD and lower in neovascular AMD. Authors interpret that as what they call a “paradoxical” protective role of ApoB-100—as the nAMD patients with higher ApoB levels respond better to the anti-VEGF therapy and have smaller CNVs in general, the authors suspect that higher ApoB levels lead to the formation of drusen and non-neovascular AMD but protect from progression to nAMD. In another study by Curcio et al., ApoB-100 was associated with the progression of dry AMD to its neovascular form in a response-to-retention pattern that was similar to that of coronary artery disease [55,56]. In a large genome-wide association study (GWAS), the ApoB level was associated with a lower risk of intermediate and geographic atrophy (GA) AMD, but not with the presence of CNV [57].

APOA2 and apoB-100 were both up-regulated in the AMD group in our study, which is in line with other studies, where elevated levels of APOA were also observed in serum [58] or in Bruch’s membrane (BM) (apoB-100) [49,59].

### 4.5. Inflammation and Complement Proteins

The concept of a prolonged inflammatory process and its detrimental effect on the ability of RPE cells to “self-clean” have been well documented in numerous studies. With aging, the RPE cells lose their ability to eliminate the metabolic products, which leads to the accumulation of the multicomponent debris between the BM and the RPE cells. Both the toxic debris itself and the sheer physical pressure that it exerts on the RPE cells lead to increased local inflammation. While a transient inflammatory process is beneficial for the tissue, when prolonged, it overpowers the cells’ ability to regenerate and leads to the collapse of the regenerative process [3,60,61,62]. This, in turn, results in cell death and the distortion of the natural blood–retinal barrier, choroidal neovascularization ingrowth and exudation with a hemorrhage—the hallmarks of nAMD. Inflammation, together with retinal autoantibodies, seems to play an important role in neovascularization, as has been discussed in great detail in a paper by Heloterä et al. [3]. In this study, we identified numerous alterations in the inflammatory-related proteins, associated mainly with complement activation and reactive oxygen species. Also, we noticed significant alterations in the levels of acute phase proteins (APPs). APPs can be roughly divided into positive (increasing inflammation) and negative (decreasing inflammation). The positive APPs that we identified were fibrinogen (down-regulated), complement factors (up-regulated), alpha-1-antitrypsin (down-regulated), and alpha-1-acid glycoprotein 1 (up-regulated), while the negative APPs were albumin (up-regulated) and RBP4 (up-regulated). This is somewhat contrary to the concept of AMD as a disease where persistent inflammation occurs. On the other hand, all the patients were receiving anti-VEGF injections, which may have altered their blood parameters, similarly to the down-regulation of the coagulation cascade proteins.

Properdin is a potent inflammation mediator, an activator of an alternative complement pathway of complement, which is commonly linked with AMD’s pathogenesis. Properdin is also a pattern-recognition protein, involved in apoptotic and necrotic pathways [63]. It seems that the over-activation of complementation plays a crucial role in AMD’s development and progression [64,65]. One of the factors in the alternative pathway that is most commonly associated with AMD is complement factor H (CFH) [60,66,67]. Properdin was down-regulated in our study in the AMD group.

Hemopexin (HPX) acts as a protective protein by binding heme—an inflammation-amplifying enzyme originating from hemoglobin after erythrocyte lysis [68,69]. Heme toxicity is a serious issue, especially in large subretinal hemorrhages, resulting in irreversible macular scarring [70]. HPX is also associated with CFH and thus complements activation. The HPX plasma levels corresponded with three loci of CFH genes in pQTL analysis [71], but these findings were not confirmed in the following study by Lauwen et al., where no correlation with CFH, nor the up-regulation of HPX in the blood plasma of AMD patients, was observed [72]. In our study, HPX showed more than two-fold up-regulation in the AMD group. Higher HPX levels can be related to the lowered coagulation balance proteins in the AMD group, acting in a counterbalancing protective manner.

Plasma protease C1 inhibitor (C1INH) is a potent anti-inflammatory protein from the serpin family. Its anti-inflammatory effect, besides from the protease inhibition, also derives from interaction with endothelial cells end leukocytes, mitigating leukocyte migration [73]. In a large study focused on this particular protein, C1INH was significantly up-regulated in the plasma of AMD patients [74], which correlates with our findings of over two-fold up-regulation of C1INH.

AGP is a plasma protein involved in binding and transportation processes. It is also one of the acute phase proteins [75]. AGP was previously described in proteomic studies concerning AMD as being up-regulated in dry AMD [27] and identified in both the vitreous body and aqueous humor in neovascular AMD [25,76].

Complement C1q subcomponent subunit B was also up-regulated almost three-fold in our study.

Albumin, the most abundant circulatory protein, which was found to be two-fold up-regulated in the AMD group, lately became a target in the anti-inflammatory treatment of AMD. The heme–albumin complex was recently proven to reduce the detrimental effect of reactive oxygen species on the retina in the ARPE-19 cell culture [77].

### 4.6. Limitations of the Study

Our study has several limitations. Most notably, the relatively small sample size of our study group should be mentioned. However, given the lack of similar studies concerning the Central European population, we contribute some further data to the field of proteomics. Another limitation to consider is the uncertainty regarding the health status of the patients in our study. The presence of co-existing diseases was excluded based on medical records and by patients’ family doctors, but a full medical examination was not conducted before collecting the samples for the study. This is particularly important due to the potential disturbances in the proteomic study’s results caused by common systemic diseases such as diabetes or hyperlipidemia [78,79,80].

## 5. Conclusions

In this study, we identified 17 different proteins in the blood serum of nAMD patients. In addition to local tissue alterations, our results indicate that systemic biomarkers link nAMD to a changed cellular transport mechanism, lipid metabolism, extracellular matrix re-organization, coagulation processes and inflammatory responses. Thus, proteomics, together with other omic analysis, may open new ways to understand AMD’s pathology and its progression and to analyze treatment responses.

## Figures and Tables

**Table 1:** Up-regulated serum proteins. Ratio (Rt)—a parameter indicating the overexpression or suppression of a protein. Rt shows how many times the protein content is up- or down-regulated compared to the control group. Score is the probability that the result is not random. A higher score indicates a more confident match. Accession number refers to the Uniprot human database.

Name	Accession Number	Entry Name	Ratio	Score
RET4_HUMAN	P02753	Retinol-binding protein 4	1.00:0.27	200
ITIH2_HUMAN	P19823	Inter-alpha-trypsin inhibitor heavy chain H2	1.00:0.27	137.34
C1QB_HUMAN	P02746	Complement C1q subcomponent subunit B	1.00:0.36	118.75
APOA2_HUMAN	P02652	Apolipoprotein A-II	1.00:0.36	117.69
ITIH1_HUMAN	P19827	Inter-alpha-trypsin inhibitor heavy chain H1	1.00:0.26	101.39
HEMO_HUMAN	P02790	Hemopexin	1.00:0.40	84.1
APOB_HUMAN	P04114	Apolipoprotein B-100	1.00:0.40	71.24
A1AG1_HUMAN	P02763	Alpha-1-acid glycoprotein 1	1.00:0.26	70.02
IC1_HUMAN	P05155	Plasma protease C1 inhibitor	1.00:0.44	61.55
ALBU_HUMAN	P02768	Albumin	1.00:0.49	59.92
ZPI_HUMAN	Q9UK55	Protein Z-dependent protease inhibitor	1.00:0.46	48.72

**Table 2:** Down-regulated serum proteins. Ratio (Rt)—a parameter indicating the overexpression or suppression of a protein. Rt shows how many times the protein content is up- or down-regulated compared to the control group. Score is the probability that the result is not random. A higher score indicates a more confident match. Accession number refers to the Uniprot human database.

Name	Accession Number	Entry Name	Ratio	Score
THRB_HUMAN	P00734	Prothrombin	1.00:2.61	131.67
PROP_HUMAN	P27918	Properdin	1.00:2.23	98.24
F13A_HUMAN	P00488	Coagulation factor XIII A chain	1.00:3.51	72.72
A1AT_HUMAN	P01009	Alpha-1-antitrypsin	1.00:2.66	48.01
TRY1_HUMAN	P07477	Trypsin-1	1.00:2.52	44.2
FIBA_HUMAN	P02671	Fibrinogen alpha chain	1.00:2.07	39.49

**Table 3:** Main functions of significantly differing proteins according to STRING database. Protein name refers to the Uniprot human database.

Protein Name	STRING Abbreviation	Function According to STRING
RET4_HUMAN	RBP4	Transporting retinol from liver to tissues
ITIH2_HUMAN	ITIH2	Binding agent between hyaluronan and serum proteins
ITIH1_HUMAN	ITIH1	Binding agent between hyaluronan and serum proteins
C1QB_HUMAN	C1QB	Cooperation with the proenzymes C1r and C1s to yield C1
APOA2_HUMAN	APOA2	Taking part in HDL metabolism and its stabilization
APOB_HUMAN	APOB	Recognition signal for the cellular binding and internalization of LDL particles by the apo-B/E receptor
HEMO_HUMAN	HPX	Taking part in heme binding and transporting it to the liver
A1AG1_HUMAN	ORM1	Transporting numerous ligands in the blood stream
IC1_HUMAN	IC1	Probably taking part in blood coagulation, fibrinolysis and the generation of kinins
ALBU_HUMAN	ALB	Binding ions such as Ca(II), Na(I), K(I) and also particles like fatty acids, hormones, bilirubin and drugs
ZPI_HUMAN	SERPINA10	Together with PROZ, calcium and phospholipids, blocking coagulation protease factor Xa
THRB_HUMAN	F2	Active in inflammation and wound healing
PROP_HUMAN	CFP	Taking part in CFI-CFH-mediated degradation of Complement C3 beta chain (C3b)
F13A_HUMAN	F13A1	Stabilizing the fibrin clot
A1AT_HUMAN	SERPINA 1	Inactivating of serine proteases
TRY1_HUMAN	PRSS1	Belongs to the peptidase S1 family
FIBA_HUMAN	FGA	Basic component of blood clots

## Data Availability

All data are available from the corresponding author.
